# Nationwide trends and disparities in deaths following cardiogenic shock and sepsis in the United States (1999–2023): insights from the CDC WONDER database

**DOI:** 10.1186/s12872-025-05402-3

**Published:** 2025-12-06

**Authors:** Nafila Zeeshan, Areej Iftikhar, Laiba Sultan, Sahar Ahsan, Amna Parvez, Muhammad Ali, Hamayel Qadir, Rumman Javed, Shahreena Athar Siddiqui, Kamil Ahmad Kamil

**Affiliations:** 1 Department of Medicine, Dow University of Health Sciences, Karachi, Pakistan; 2 Department of Medicine, Allama Iqbal Medical College, Lahore, Pakistan; 3 Department of Medicine, Khawaja Muhammad Safdar Medical College, Sialkot, Pakistan; 4 Liaquat National Hospital and Medical College, Karachi, Pakistan; 5 Dow International Medical College, Karachi, Pakistan; 6 Department of Medicine, Ayub Medical College, Abbottabad, Pakistan; 7 Department of Internal Medicine, Mirwais Regional Hospital, Kandahar, Afghanistan

**Keywords:** Cardiogenic shock, Sepsis, CDC WONDER, Mortality, united states, Epidemiology

## Abstract

**Background:**

Cardiogenic shock (CS) with sepsis is a highly fatal condition, yet national mortality trends and demographic disparities in these co-occurring conditions remain poorly characterized.

**Methods:**

We analyzed CDC WONDER Multiple Cause of Death data (1999–2023) to identify adult deaths involving both CS and sepsis. Age-adjusted mortality rates (AAMRs), crude mortality rates (CMRs), average annual percent change (AAPC), and annual percent change (APC) by period were calculated and stratified by sex, race, age, census region, urbanization, and state.

**Results:**

From 1999 to 2023, 59,898 deaths were recorded, overwhelmingly in inpatient medical facilities (94.61%). The national AAMR rose from 0.65 (1999) to 2.01 (2023), with an overall AAPC of 5.39 (95% CI: 4.24–6.56, *p* < 0.001). Mortality was higher in males (AAMR: 1.28; AAPC: 5.20) compared to females (AAMR: 0.78; AAPC: 5.35). NH Black or African American individuals had the highest AAMR (1.69), followed by Hispanic (0.98), and NH White (0.93). Older adults had the greatest CMR (3.51), compared with middle-aged (0.77) and young adults (0.12). By region, AAMR was highest in the South (1.06) and Northeast (1.05), lower in the Midwest (0.84), and lowest in the West (0.10). Non-metropolitan areas had higher AAMR (0.89; AAPC: 6.34) compared with metropolitan areas (0.86; AAPC: 4.78). States with the highest burden included Rhode Island, North Carolina, West Virginia, and Connecticut.

**Conclusion:**

Mortality from CS with sepsis increased significantly over two decades, with pronounced disparities across sex, race, age, and geography. These findings highlight the urgent need for targeted national strategies.

**Supplementary Information:**

The online version contains supplementary material available at 10.1186/s12872-025-05402-3.

## Introduction

Cardiogenic shock (CS), a highly fatal condition, is a significant cause of mortality and morbidity in the United States (U.S.) [1] According to the National Inpatient Sample (NIS) database, hospitalizations attributed to CS have increased by a factor of three from 2004 to 2018, highlighting the healthcare burden [2]. Although mechanical circulatory support (MCS) strategies, timely revascularization, and advances in intensive care have contributed to the reduction observed in mortality in patients with CS, the 1-year mortality rate for CS remains between 30 and 50% [3–5]. Moreover, CS also accounts for a substantial economic burden, with median one-year healthcare costs reaching roughly $45,700 among patients who survived at least to discharge [6].

Similarly, sepsis is a major health burden involving organ dysfunction that results from an uncontrollable host response to infection, accounting for 20% of mortalities worldwide [7]. Sepsis contributes to at least 1.7 million hospitalizations of US adults each year, with approximately $62 billion financial burden annually [8, 9].

Studies have shown that sepsis and CS coexist, causing high mortality and complicated presentation. A U.S. sample of over 444.000 AMI-CS admissions from 2000 to 2014 shows about 6% had concomitant sepsis, with higher rates of non-cardiac organ failure [10]. Pathophysiology includes systemic inflammation, vasoplegia, and septic cardiomyopathy caused by sepsis, which amplifies low cardiac output and end-organ hypoperfusion mediated by CS, leading to hemodynamic collapse and multi-organ failure [11].

Despite growing evidence of pathophysiological and clinical associations between the two conditions, limited research has examined national mortality trends using death certificate data where both CS and sepsis have been recorded as contributing causes of death. To address this gap, this study aims to examine CS and sepsis-related mortality patterns among U.S. adults aged 25 and older using nationally representative data from CDC WONDER. Temporal patterns and variations by sex, geographic region, and urban-rural classification were included to allow for comparative analysis and to support informed public health and policy making.

## Materials and methods

### Study setting and population

This study analyzed mortality records from the Centers for Disease Control and Prevention (CDC) Wide-ranging Online Data for Epidemiologic Research (WONDER) Multiple Cause of Death database [12, 13]. Publicly available mortality data about individuals in the United States was extracted from 1999 to 2023, which listed both cardiogenic shock and sepsis as the contributing cause of death. Mortality abstraction was carried out using the International Classification of Diseases, 10th Revision (ICD-10) codes. Specifically, CS was identified using the code R57.0 (CS), and Sepsis was identified using A02.1 (Salmonella Sepsis), A20.7 (Septicaemic Plague), A22.7 (Anthrax Sepsis), A26.7 (Erypsipelothrix Sepsis), A32.7 (Listerial Sepsis), A40.0 (Streptococcal Septicaemia) to A41 (Other Septicaemia) and B37.7 (Candidal Sepsis). Additional demographic and epidemiologic variables, including age, sex, race/ethnicity, urban-rural classification, and year of death, were also extracted for analysis. As the dataset is publicly available and de-identified, Institutional Board Review (IRB) approval was not required. All study methods adhered to the Strengthening the Reporting of Observational Studies in Epidemiology (STROBE) guidelines.

### Data extraction

Demographic variables such as age, sex, race/ethnicity, census region, and place of death were categorized using standardized classifications. Race and ethnicity were grouped into Hispanic and non-Hispanic populations, with the latter further divided into White, Black or African American, American Indian or Alaska Native, and Asian or Pacific Islander categories. Age cohorts included Young adults (25–44 years), Middle-aged adults (45–64 years) and Older adults (≥ 65 years old). Geographic regions were classified according to the U.S. Census Bureau’s regional framework, which designates four regions: Northeast, Midwest, South, and West. Urban–rural status was determined using the National Center for Health Statistics (NCHS) Urban–Rural Classification Scheme [14]. Rural areas were defined as those with populations under 50,000 and were further classified as Micropolitan (counties in micropolitan statistical areas) or Non-Core (counties not meeting micropolitan criteria). Urban areas were subdivided into four categories: Large Central Metropolitan (counties within metropolitan areas of at least one million residents that include at least 250,000 residents of a principal city), Large Fringe Metropolitan (counties in metropolitan areas of one million or more that are not designated as Large Central), Medium Metropolitan (counties in metropolitan areas with populations between 250,000 and 999,999), and Small Metropolitan (counties in metropolitan areas with populations between 50,000 and 249,999) (14).

### Statistical analysis

National trends in mortality attributable to both CS and sepsis were analyzed using both crude mortality rates (CMRs) and age-adjusted mortality rates (AAMRs), expressed per 100,000 population, for the years 1999 through 2023. CMRs were calculated by dividing the number of deaths attributed to CS and sepsis by the corresponding U.S. population for each year. AAMRs were computed using the direct standardization method to the 2000 U.S. population, allowing for comparisons across different time periods while accounting for variations in age distribution.

The Joinpoint regression program (Version 5.2.0, National Cancer Institute) was used to assess changes in mortality rates over time [15]. Annual percent change (APC) in AAMRs, along with corresponding 95% confidence intervals (CIs), was determined by applying log-linear regression models to detect significant temporal shifts. Two-tailed t-tests were employed to determine whether the APCs indicated a statistically significant increase or decrease based on whether the slope differed from zero. Additionally, we calculated the average annual percent change (AAPC) and its 95% CI by fitting a regression line to the natural logarithm of the rates. A test for parallelism was also conducted to assess whether the trends differed significantly between the two cohorts (e.g., mortality involving both CS and sepsis), stratified by variables such as sex, race, census region, and urban-rural classification. A p-value of less than 0.05 was considered statistically significant.

## Results

From 1999 to 2023, there were 59,898 deaths due to CS and sepsis (Tables 1 and 2). Between 1999 and 2023, most deaths occurred in inpatient medical facilities, accounting for 94.61% of all deaths. The remaining deaths were distributed among other locations, with outpatient medical facilities (2.34%), followed by nursing homes/long-term care facilities (1.04%), hospice facilities (0.76%), the decedent’s home (0.61%), other locations (0.34%), and unknown locations (0.22%). Medical facilities with dead-on arrival and medical facilities with status unknown had smaller proportions, as both accounted for 0.04% of deaths (Supplemental Table 7).

**Table 1:** National cardiogenic shock and Sepsis-related deaths stratified by sex and race, 1999–2023

Year	Deaths								
	Overall (Cardiogenic Shock and Sepsis Combined)	Cardiogenic Shock (Alone)	Sepsis (Alone)	Female	Male	NH-Black or African American	NH-White	Hispanics or Latinos	Population
1999	1,135	18,946	137,394	549	586	190	910	73	180,408,769
2000	1,035	17,516	135,855	496	539	163	842	66	181,984,640
2001	984	15,816	138,370	469	515	172	786	66	184,305,128
2002	1,079	15,379	141,583	519	560	160	882	75	186,208,028
2003	1,016	14,395	143,754	472	544	142	828	62	188,090,429
2004	1,072	14,120	144,104	533	539	156	878	67	190,205,384
2005	1,109	13,729	150,905	532	577	168	887	74	192,551,384
2006	1,108	13,763	150,766	538	570	174	892	77	195,019,359
2007	1,142	13,698	150,844	542	600	183	912	91	197,403,777
2008	1,297	14,458	157,630	597	700	191	1,049	100	199,795,090
2009	1,311	14,330	155,788	603	708	225	1,038	90	202,107,016
2010	1,419	14,930	158,097	614	805	203	1,152	105	203,891,983
2011	1,552	15,875	163,419	706	846	264	1,221	114	206,592,936
2012	1,709	16,858	163,765	770	939	291	1,337	146	208,826,037
2013	2,013	17,948	172,318	861	1,152	329	1,597	132	211,085,314
2014	2,359	19,981	180,905	1,048	1,311	430	1,826	168	213,809,280
2015	2,778	22,518	193,872	1,207	1,571	475	2,166	224	216,553,817
2016	3,208	24,676	196,631	1,333	1,875	529	2,518	264	218,641,417
2017	3,461	26,589	202,446	1,432	2,029	602	2,680	341	221,447,331
2018	3,870	29,040	204,711	1,579	2,291	676	2,987	357	223,311,190
2019	4,104	31,503	199,984	1,664	2,440	749	3,164	378	224,981,167
2020	4,662	33,936	241,176	1,910	2,752	838	3,562	503	226,635,013
2021	5,422	38,452	259,995	2,183	3,239	969	4,160	581	228,238,412
2022	5,501	38,940	242,515	2,302	3,199	1,006	4,164	599	229,508,599
2023	5,552	39,401	224,337	2,293	3,259	1,057	4,150	585	23,152,962
Total	59,898	536,797	4,411,164	25,752	34,146	10,342	46,588	5,338	5,163,131,262

**Table 2:** Annual percent change (APC) and average annual percent change (AAPC) in cardiogenic shock and sepsis-related mortality

Category	Segment	APC (95% CI)	*p*-value	AAPC (95% CI)	*p*-value
Overall (Cardiogenic Shock and Sepsis Combined)	1999–2009	0.41 (−1.26 to 2.10)	0.616	5.39 (4.24 to 6.56)	< 0.001
	2009–2021	10.68 (9.60 to 11.78)	< 0.001		
	2021–2023	0.08 (−9.30 to 10.44)	0.986		
Cardiogenic Shock (Alone)	1999–2004	−7.70 (−8.86 to −6.53)	< 0.001	1.25 (0.75 to 1.75)	< 0.001
	2004–2011	−0.35 (−1.38 to 0.69)	0.485		
	2011–2021	7.34 (6.88 to 7.81)	< 0.001		
	2021–2023	0.73 (−2.96 to 4.56)	0.681		
Sepsis (Alone)	1999–2018	0.10 (−0.16 to 0.36)	0.427	0.33 (−0.73 to 1.40)	0.545
	2018–2021	8.14 (0.27 to 16.63)	< 0.001		
	2018–2023	−8.38 (−14.84 to −1.42)	< 0.001		
By Sex					
Female	1999–2010	0.49 (−1.16 to 2.17)	0.54	5.35 (3.78 to 6.94)	< 0.001
	2010–2015	13.01 (6.04 to 20.44)	< 0.001		
	2015–2023	7.59 (5.96 to 9.24)	< 0.001		
Male	1999–2004	−3.22 (−6.60 to 0.27)	0.067	5.20 (3.84 to 6.58)	< 0.001
	2004–2011	3.98 (1.46 to 6.56)	0.005		
	2011–2016	14.03 (10.22 to 17.97)	< 0.001		
	2016–2021	9.03 (6.39 to 11.74)	< 0.001		
	2021–2023	0.94 (−5.53 to 7.86)	0.761		
By Race					
NH-Black or African American	1999–2003	−8.58 (−13.57 to −3.30)	0.004	4.64 (3.26 to 6.05)	< 0.001
	2003–2010	3.76 (0.76 to 6.84)	0.017		
	2010–2021	10.67 (9.73 to 11.62)	< 0.001		
	2021–2023	3.80 (−3.52 to 11.68)	0.292		
NH-White	1999–2001	−6.86 (−25.24 to 16.03)	0.499	4.88 (2.70 to 7.10)	< 0.001
	2001–2009	1.25 (−1.63 to 4.22)	0.371		
	2009–2021	10.50 (9.40 to 11.61)	< 0.001		
	2021–2023	−0.60 (−10.32 to 10.16)	0.901		
Hispanics or Latinos	1999–2011	−1.25 (−3.62 to 1.18)	−1.09	4.36 (2.48 to 6.28)	4.88 (2.70 to 7.10)
	2011–2021	13.03 (10.64 to 15.47)	12.08		
	2021–2023	−2.44 (−16.04 to 13.37)	−0.35		
By Census Region					
Northeast	1999–2001	−11.81 (−27.39 to 7.11)	0.187	4.07 (2.00 to 6.19)	< 0.001
	2001–2012	2.53 (1.00 to 4.07)	0.003		
	2012–2016	12.63 (4.47 to 21.43)	0.004		
	2016–2023	6.78 (5.15 to 8.43)	< 0.001		
Midwest	1999–2006	−2.17 (−4.73 to 0.45)	0.097	5.38 (3.90 to 6.87)	< 0.001
	2006–2012	7.12 (2.70 to 11.73)	0.004		
	2012–2020	11.93 (10.04 to 13.86)	< 0.001		
	2020–2023	3.26 (−1.50 to 8.24)	0.167		
South	1999–2009	−0.78 (−2.17 to 0.63)	0.255	5.16 (3.91 to 6.42)	< 0.001
	2009–2015	15.01 (11.45 to 18.69)	< 0.001		
	2015–2021	8.14 (5.89 to 10.43)	< 0.001		
	2021–2023	−1.19 (−8.87 to 7.15)	0.756		
West	1999–2009	1.10 (−1.62 to 3.90)	0.413	6.53 (5.27 to 7.81)	< 0.001
	2009–2023	10.59 (9.54 to 11.65)	< 0.001		
By Urbanization					
Urban	1999–2003	−3.68 (−6.99 to −0.25)	0.039	4.79 (2.96 to 6.64)	< 0.001
	2003–2009	2.28 (−0.15 to 4.76)	0.063		
	2009–2012	6.52 (−3.37 to 17.43)	0.173		
	2012–2015	15.16 (6.26 to 24.80)	0.004		
	2015–2020	7.97 (6.52 to 9.45)	< 0.001		
Rural	1999–2008	−0.63 (−3.25 to 2.06)	0.617	6.34 (4.59 to 8.12)	< 0.001
	2008–2016	13.75 (10.51 to 17.08)	< 0.001		
	2016–2020	8.26 (3.34 to 13.42)	0.003		
By Age Group					
Young Adults	1999–2007	3.63 (−1.49 to 9.02)	0.156	8.13 (5.71 to 10.62)	< 0.001
	2007–2021	12.65 (10.97 to 14.35)	< 0.001		
	2021–2023	−3.71 (−19.93 to 15.79)	0.67		
Middle-Aged Adults	1999–2007	−0.51 (−3.31 to 2.36)	0.708	6.50 (5.11 to 7.91)	< 0.001
	2007–2021	12.17 (11.21 to 13.14)	< 0.001		
	2021–2023	−2.71 (−12.92 to 8.69)	0.608		
Older Adults	1999–2009	−0.19 (−1.69 to 1.33)	0.794	4.68 (3.63 to 5.73)	< 0.001
	2009–2021	9.50 (8.50 to 10.50)	< 0.001		
	2021–2023	1.36 (−7.37 to 10.92)	0.755		

### Annual trends

#### Cardiogenic shock

**Fig. 1:**
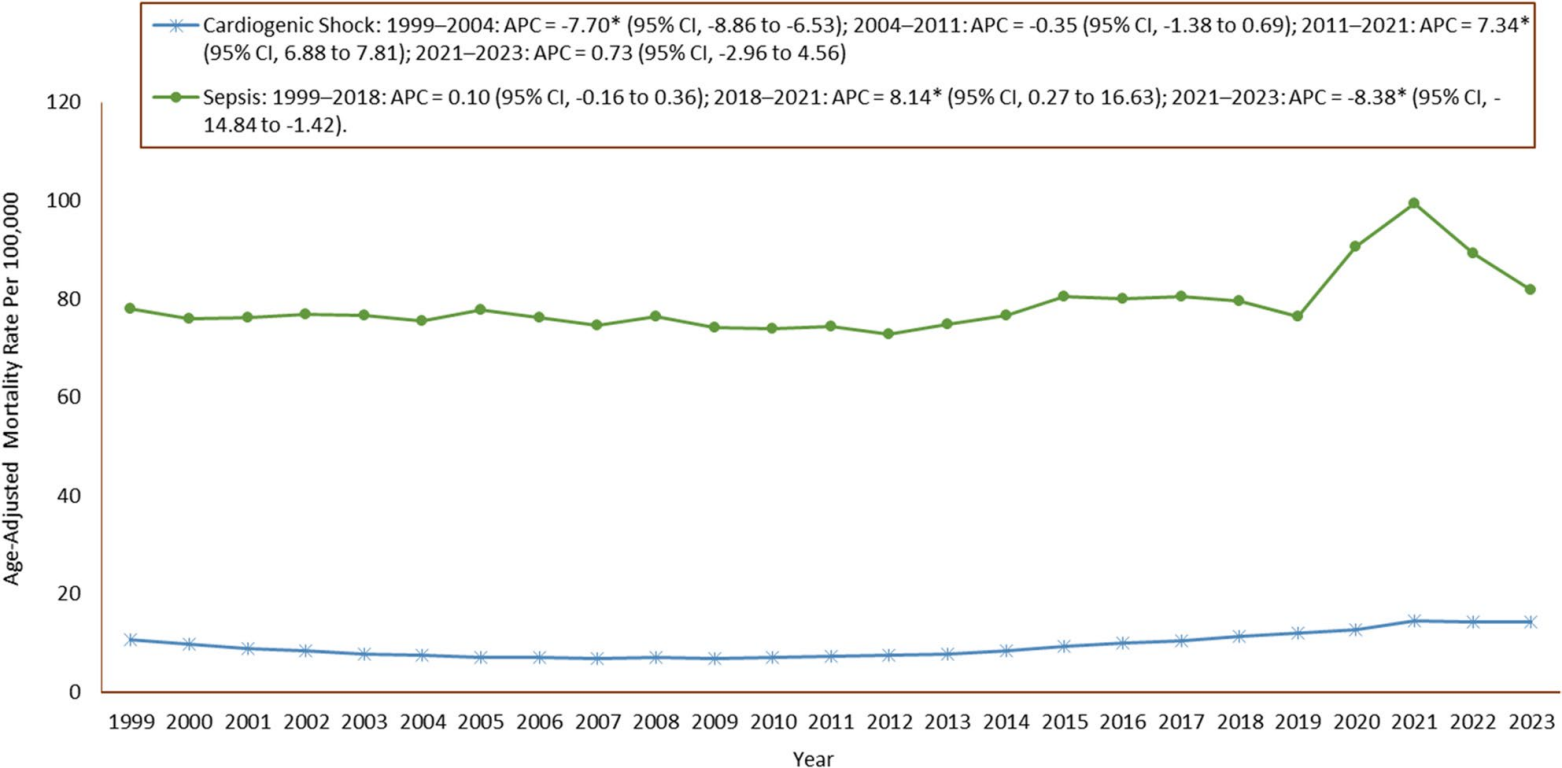
Annual AAMRs for Cardiogenic Shock and Sepsis alone, United States, 1999–2023 APC = Annual Percentage Change, CI = Confidence Interval *Indicates that the Annual Percentage Change (APC) is significantly different from zero at α = 0.05

A total of 536,797 CS-related deaths were recorded among adults ≥ 25 years. The AAMR increased from 10.72 (95% CI: 10.57–10.87) in 1999 to 14.23 (95% CI: 14.08–14.37) in 2023. Over the full period, AAPCs increased (AAPC: 1.25; 95% CI: 0.75–1.75). The trend showed a U-shaped pattern, with a significant decrease from 1999 to 2004 (APC: −7.70; 95% CI: −8.86 to − 6.53), followed by an insignificant decrease from 2004 to 2011 (APC: −0.35; 95% CI: −1.38 to 0.69). This reversed into a significant rise from 2011 to 2021 (APC: 7.34; 95% CI: 6.88 to 7.81), ending with an insignificant increase from 2021 to 2023 (APC: 0.73; 95% CI: −2.96 to 4.56) **(**Tables 1 and 2; Fig. 1, Supplementary Table 1).

#### Sepsis

Sepsis accounted for 4,411,164 deaths. The AAMR increased from 77.83 (95% CI: 77.42–78.24) in 1999 to 81.79 (95% CI: 81.44–82.13) in 2023. Overall, the trend was not statistically significant (AAPC: 0.33; 95% CI: −0.73 to 1.40). From 1999 to 2018, AAMRs increased insignificantly (APC: 0.10; 95% CI: −0.16 to 0.36), followed by a significant rise from 2018 to 2021 (APC: 8.14; 95% CI: 0.27 to 16.63). This shifted to a significant decline from 2021 to 2023 (APC: −8.38; 95% CI: −14.84 to − 1.42) **(**Tables 1 and 2; Fig. 1, Supplementary Table 1).

#### Cardiogenic shock and sepsis combined

**Fig. 2:**
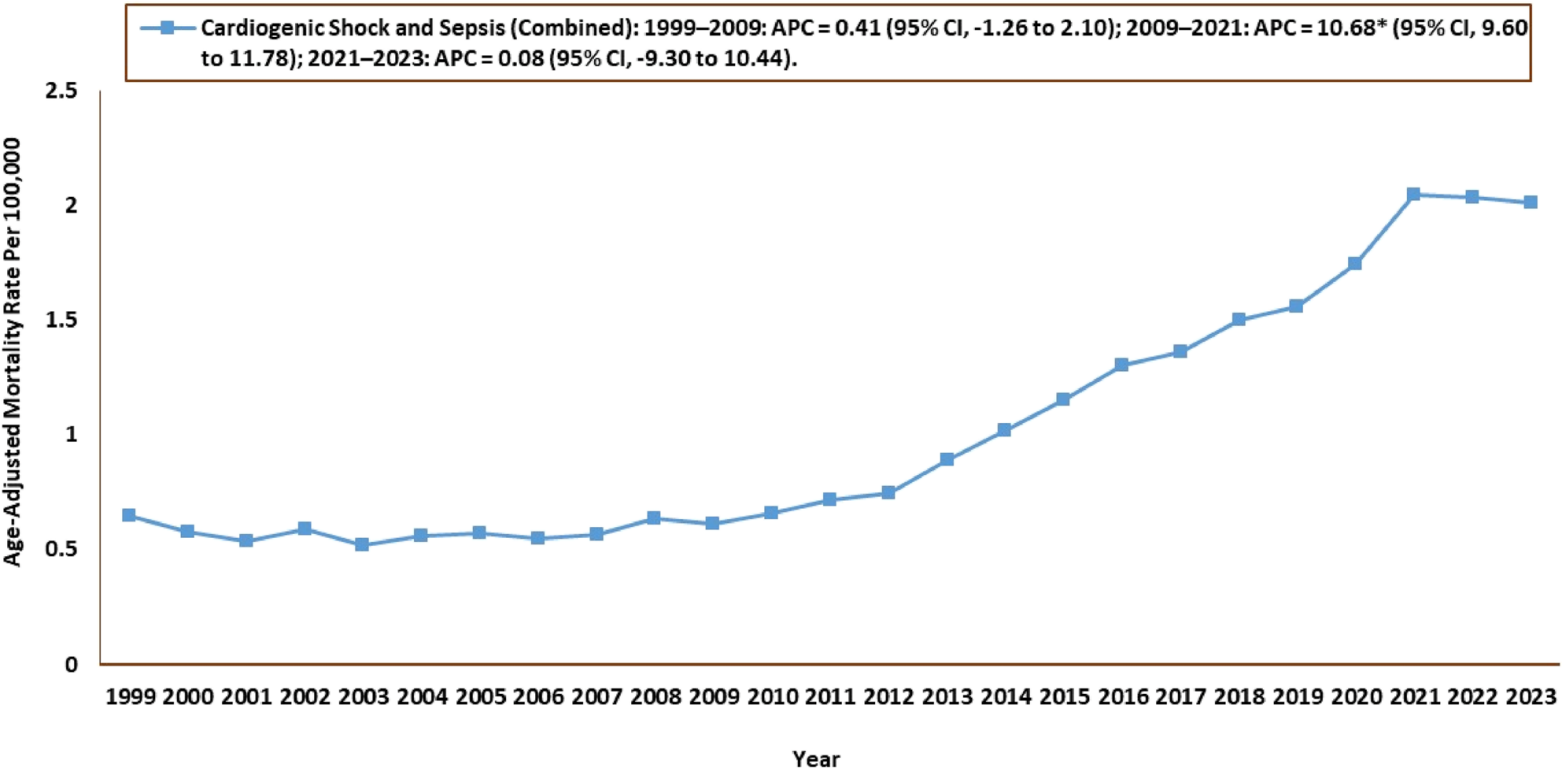
Cardiogenic Shock and Sepsis–Related Age-Adjusted Mortality Rates, 1999–2023 APC = Annual Percentage Change, CI = Confidence Interval *Indicates that the Annual Percentage Change (APC) is significantly different from zero at α = 0.05

The AAMRs for CS and sepsis-related deaths in individuals were 0.65 in 1999 (95% CI: 0.61–0.69) and rose to 2.00 in 2023 (95% CI: 1.96–2.06). During the overall study period from 1999 to 2023, AAPCs increased (AAPC: 5.39; 95% CI, 4.24 to 6.56). The overall AAMR experienced a consistent increase throughout the study period, increasing insignificantly from 1999 to 2009 (APC: 0.41; 95% CI: −1.26 to 2.10) and then significantly till 2021 (APC: 10.7; 95% CI: 9.60 to 11.8). This followed an insignificant increase till 2023 (APC: 0.08; 95% CI: −9.30 to 10.4) **(**Tables 1 and 2; Fig. 2, Supplementary Table 2).

### Sex-Specific trends

**Fig. 3:**
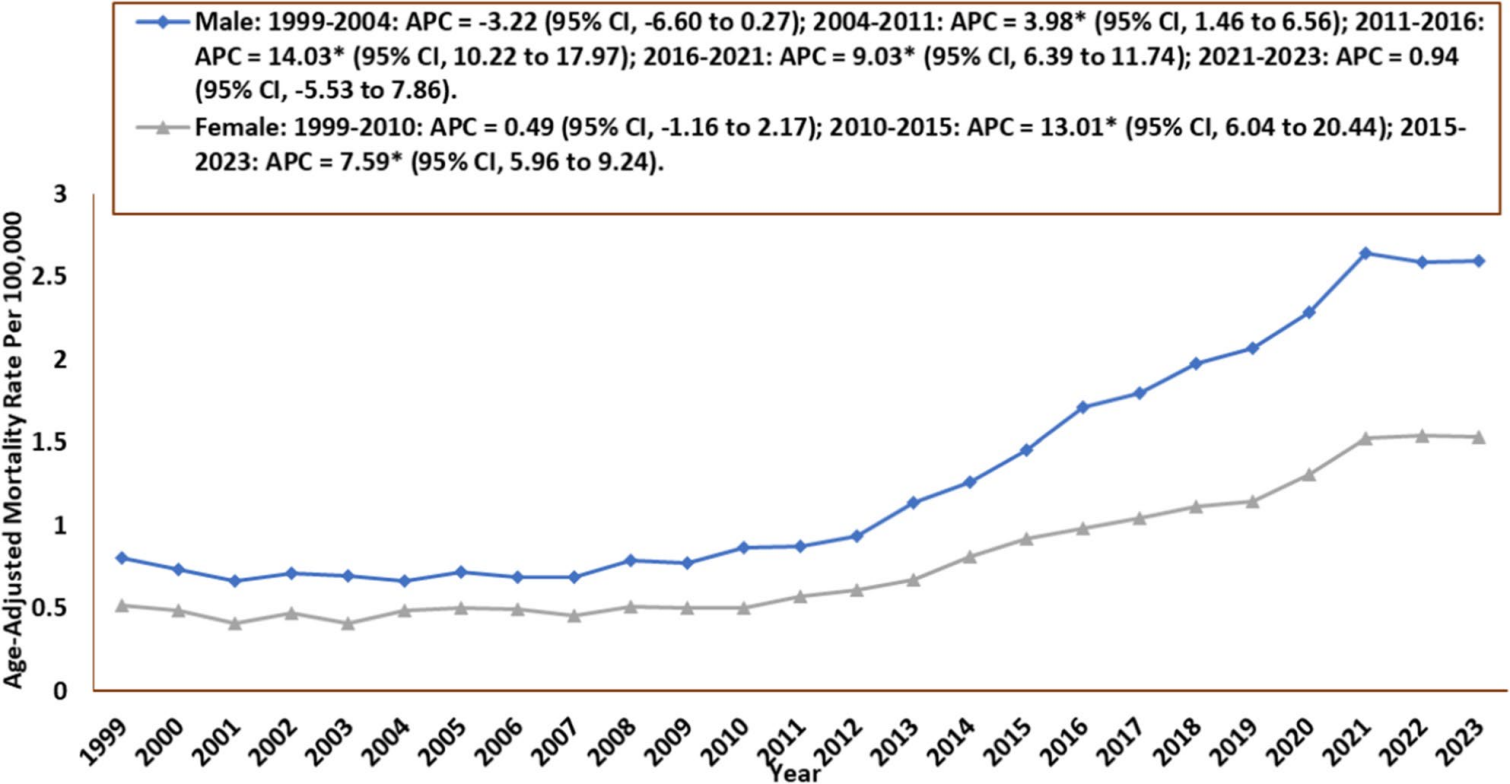
Cardiogenic Shock and Sepsis–Related Age-Adjusted Mortality Rates by Sex, 1999–2023 APC = Annual Percentage Change, CI = Confidence Interval *Indicates that the Annual Percentage Change (APC) is significantly different from zero at α = 0.05

Men experienced a higher total number of deaths (34,146; 57%) than women (25,752; 43%). For females, the AAMR was 0.52 in 1999 (95% CI: 0.47–0.56) and rose to 1.53 in 2023 (95% CI: 1.47–1.60). The AAMR for females increased throughout the period, starting with an insignificant increase from 1999 to 2010 (APC: 0.49; 95% CI: −1.16 to 2.17), followed by a significant rise first from 2010 to 2015 (APC: 13.0; 95% CI: 6.04 to 20.4) and then till 2023 (APC: 7.59; 95% CI: 5.96 to 9.24). For males, the AAMR was 0.81 in 1999 (95% CI: 0.74 to 0.87) and increased to 2.60 in 2023 (95% CI: 2.51 to 2.69). In men, the AAMR first experienced an insignificant decrease from 1999 to 2004 (APC: −3.22; 95% CI: −6.60 to 0.27). This followed a period of consistent and significant increase between the years: 2004 and 2011 (APC: 3.98; 95% CI: 1.46 to 6.56), 2011 and 2016 (APC: 14.0; 95% CI: 10.2 to 18.0), and from 2016 to 2021 (APC: 9.03; 95% CI: 6.39 to 11.7). Finally, there was an insignificant increase till 2023 (APC: 0.94; 95% CI: −5.53 to 7.86) **(**Tables 1 and 2; Fig. 3, Supplemental Table 2).

### Race/Ethnicity specific trends

**Fig. 4:**
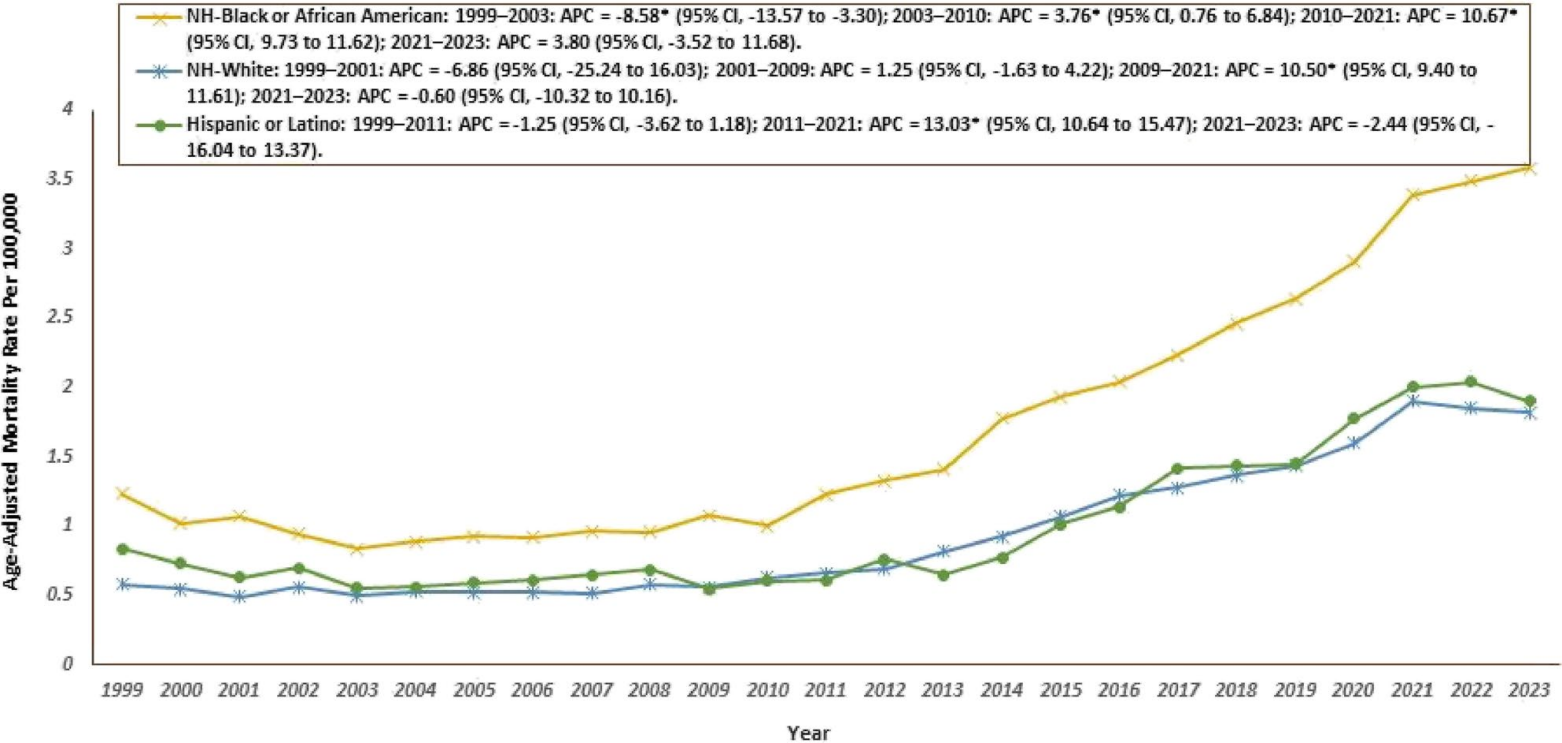
Cardiogenic Shock and Sepsis–Related Age-Adjusted Mortality Rates by Race/Ethnicity, 1999–2023 APC = Annual Percentage Change, CI = Confidence Interval *Indicates that the Annual Percentage Change (APC) is significantly different from zero at α = 0.05

Among racial and ethnic groups, Non-Hispanic (NH) Whites accounted for the largest number of deaths overall (46,588; 78%), followed by NH Black/African Americans (10,342; 17%) and Hispanic/Latinos (5,338; 9%), highlighting a substantial disparity in mortality burden across demographic categories throughout the study period. Higher AAMRs were observed among Black or African American individuals as compared to White individuals. For Black or African American individuals, the AAMR was 1.24 in 1999 (95% CI: 1.06–1.41) and increased to 3.58 in 2023 (95% CI: 3.36–3.80). The AAMR fluctuated throughout the study period, experiencing a significant decrease from 1999 to 2003 (APC: −8.58; 95% CI: −13.6 to −3.30), followed by significant increases from 2003 to 2010 (APC: 3.76; 95% CI: 0.76 to 6.84) and 2010 to 2021 (APC: 10.67; 95% CI: 9.73 to 11.62). Finally, there was an insignificant increase till 2023 (APC: 3.80; 95% CI: −3.52 to 11.68). For White individuals, the AAMR was 0.58 in 1999 (95% CI: 0.54–0.62) and increased to 1.82 in 2023 (95% CI: 1.77–1.88). The AAMR decreased insignificantly from 1999 to 2001 (APC: −6.86; 95% CI: −25.24 to 16.03) and similarly till 2009 (APC: 1.25; 95% CI: −1.63 to 4.22). From 2009 to 2021, there was a significant increase observed (APC: 10.50; 95% CI: 9.40 to 11.61) followed by an insignificant decrease till 2023 (APC: −0.60; 95% CI: −10.32 to 10.16). For Hispanic or Latino individuals, the AAMR was 0.84 in 1999 (95% CI: 0.65 to 1.06) and rose to 1.90 in 2023 (95% CI: 1.74 to 2.06). From 1999 to 2011, the rate showed a nonsignificant decline (APC: −1.25; 95% CI: −3.62 to 1.18). This was followed by a statistically significant increase from 2011 to 2021 (APC: 13.03; 95% CI: 10.64 to 15.47). In the final segment, from 2021 to 2023, there was a nonsignificant decrease (APC: −2.44; 95% CI: −16.04 to 13.37). **(**Tables 1 and 2; Fig. 4, Supplemental Table 3).

### Age-Specific trends

**Fig. 5:**
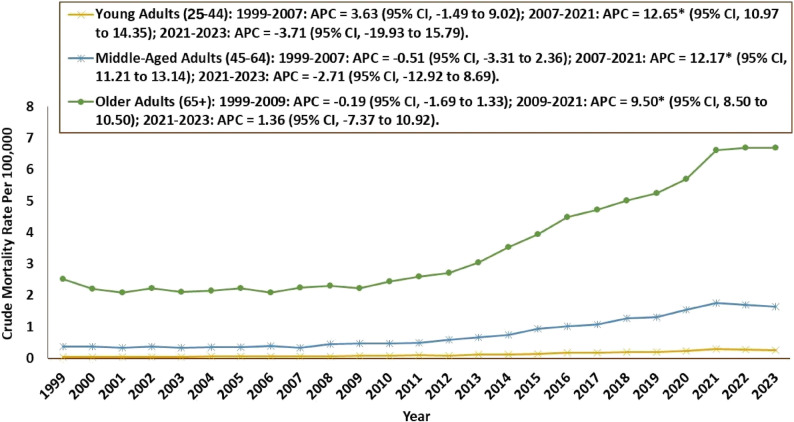
Cardiogenic Shock and Sepsis–Related Crude Mortality Rates by Age Group, 1999–2023 APC = Annual Percentage Change, CI = Confidence Interval *Indicates that the Annual Percentage Change (APC) is significantly different from zero at α = 0.05

Older adults (≥ 65 years) had the highest number of deaths (41,797; 70%), followed by middle-aged adults (45–64 years) (15,547; 26%), while young adults (25–44 years) had the fewest (2,554; 4%). For young adults, the CMR was 0.04 in 1999 (95% CI: 0.03–0.05) and rose slightly to 0.26 in 2023 (95% CI: 0.23–0.29). An insignificant rise occurred from 1999 to 2007 (APC: 3.63; 95% CI: −1.49 to 9.02), followed by a significant increase till 2021 (APC: 12.65; 95% CI: 10.97 to 14.35). Finally, there was an insignificant decrease till 2023 (APC: −3.71; 95% CI: −19.93 to 15.79). For middle-aged adults, the CMR was 0.37 in 1999 (95% CI: 0.32–0.42) and rose to 1.64 in 2023 (95% CI: 1.55–1.73). The rates decrease insignificantly from 1999 to 2007 (APC: −0.51; 95% CI: −3.31 to 2.36). This followed a period of significant increase till 2021 (APC: 12.17; 95% CI: 11.21 to 13.14), after which there was another insignificant decrease from 2021 to 2023 (APC: −2.71; 95% CI: −12.92 to 8.69). For older adults, the CMR was 2.52 in 1999 (95% CI: 2.35–2.69) and rose to 6.70 in 2023 [95% CI: 6.49–6.91]. The rate fluctuated throughout the study period, first decreasing insignificantly from 1999 to 2009 (APC: −0.19 [95% CI: −1.69 to 1.33]), and then increasing significantly from 2009 to 2021 (APC: 9.50; 95% CI: 8.50 to 10.50) and insignificantly till 2023 (APC: 1.36; 95% CI: −7.37 to 10.92]) **(**Table 2; Fig. 5, Supplementary Table 4).

### Urbanization specific trends

**Fig. 6:**
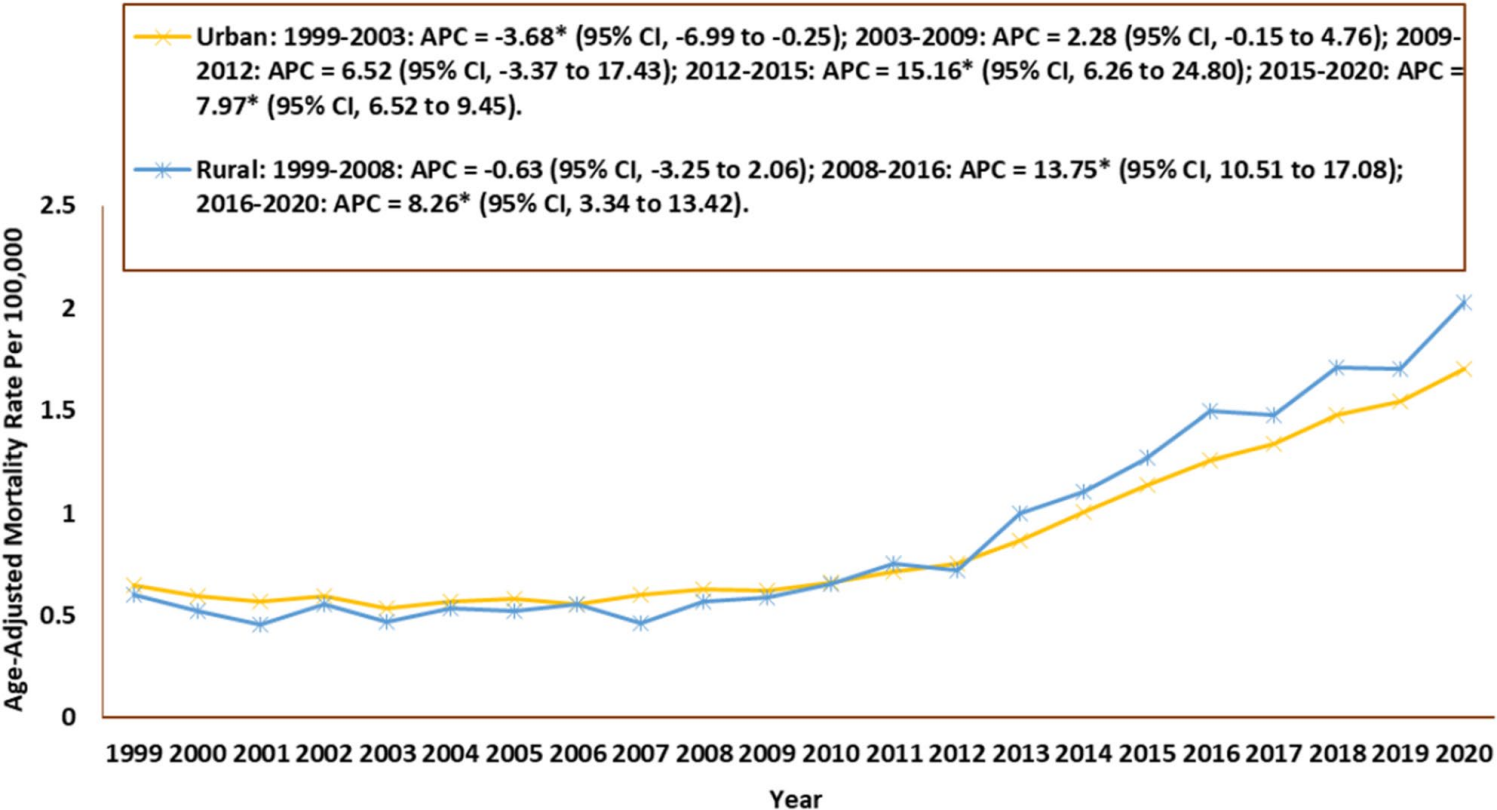
Cardiogenic Shock and Sepsis–Related Age-Adjusted Mortality Rates by Urbanization, 1999–2023 APC = Annual Percentage Change, CI = Confidence Interval *Indicates that the Annual Percentage Change (APC) is significantly different from zero at α = 0.05

Urban areas experienced a far greater number of deaths (35,709; 82%) compared to rural areas (7,714; 18%). Higher AAMRs were observed for rural areas compared to urban areas throughout the study period. For urban areas, the AAMR was 0.65 in 1999 (95% CI: 0.61–0.70) and rose to 1.70 in 2020 (95% CI: 1.65–1.76). The AAMR experienced a significant decrease from 1999 to 2003 (APC: −3.68; 95% CI: −6.99 to −0.25), followed by an increase from 2003 to 2009 (APC: 2.28; 95% CI: −0.15 to 4.76) and from 2009 to 2012 (APC: 6.52; 95% CI: −3.37 to 17.43). There was a significant rise till 2015 (APC: 15.2; 95% CI: 6.26 to 24.80) and finally from 2015 to 2020 (APC: 7.97; 95% CI: 6.52 to 9.45). For rural areas, the AAMR was 0.60 in 1999 (95% CI: 0.52–0.69) and rose to 2.03 in 2020 (95% CI: 1.88–2.17). In rural areas, the rate decreased insignificantly from 1999 to 2008 (APC: −0.63; 95% CI: −3.25 to 2.06) after which there were two significant peaks, one from 2008 to 2016 (APC: 13.75; 95% CI: 10.51 to 17.08) and one from 2016 to 2020 (APC: 8.26; 95% CI: 3.34 to 13.42) **(**Table 2; Fig. 6, Supplementary Table 5).

### Census specific trends

**Fig. 7:**
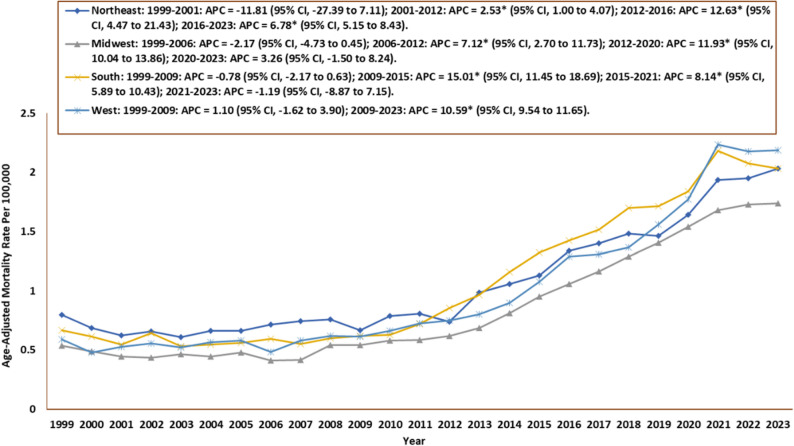
Cardiogenic Shock and Sepsis–Related Age-Adjusted Mortality Rates by Census Region, 1999–2023 APC = Annual Percentage Change, CI = Confidence Interval *Indicates that the Annual Percentage Change (APC) is significantly different from zero at α = 0.05

The South had the greatest number of deaths (30,284; 40%), followed by the West (17,224; 23%), the Northeast (14,873; 19%), and the Midwest (13,992; 18%). For Midwestern regions, the AAMR was 0.54 in 1999 (95% CI: 0.47–0.61) and increased to 1.73 in 2023 (95% CI: 1.63–1.85). The Midwest experienced an increase in average annual percent change (AAPC: 5.38; 95% CI: 3.90 to 6.87). The AAMR fluctuated throughout the study period, first decreasing insignificantly from 1999 to 2006 (APC: −2.17; 95% CI: −4.73 to 0.45) and then increasing significantly from 2006 to 2012 (APC: 7.12; 95% CI: 2.70 to 11.73) and from 2012 to 2020 (APC: 11.93; 95% CI: 10.04 to 13.86). Finally, there was an insignificant increase from 2020 to 2023 (APC: 3.26; 95% CI: −1.50 to 8.24). For Northeastern regions, the AAMR was 0.80 in 1999 (95% CI: 0.71–0.89) and rose to 2.03 in 2023 (95% CI: 1.91–2.16). The AAPC for Northeast increased from 1999 to 2020 (AAPC: 4.07; 95% CI, 2.00 to 6.19). There was an insignificant decrease in AAMR from 1999 to 2001 (APC: −11.81; 95% CI: −27.39 to 7.12) after which there were three periods of significant increases: 2001 to 2012 (APC: 2.53; 95% CI: 1.00 to 4.07), 2012 to 2016 (APC: 12.63; 95% CI: 4.47 to 21.43) and 2016 to 2023 (APC: 6.78; 95% CI: 5.15 to 8.43). For Southern areas, the AAMR was 0.67 in 1999 (95% CI: 0.60–0.74) and rose to 2.03 in 2023 (95% CI: 1.95–2.12). The AAPC for the South increased from 1999 to 2020 (AAPC: 5.16; 95% CI, 3.90 to 6.42). There was an insignificant drop in AAMR from 1999 to 2009 (APC: −0.78; 95% CI: −2.17 to 0.63). This was followed by a significant rise from 2009 to 2015 (APC: 15.01; 95% CI: 11.45 to 18.69) and 2015 to 2021 (APC: 8.14; 95% CI: 5.89 to 10.43). Finally, there was another insignificant drop between 2021 and 2023 (APC: −1.19; 95% CI: −8.87 to 7.15). For Western areas, the AAMR was 0.59 in 1999 (95% CI: 0.51–0.67) and rose to 2.19 in 2023 (95% CI: 2.07–2.30). The AAPC for the West increased the most from 1999 to 2020 (AAPC: 6.53; 95% CI: 5.27 to 7.81). AAMRs observed an insignificant drop from 1999 to 2009 (APC: 1.10; 95% CI: −1.62 to 3.90) and then a significant rise till 2023 (APC: 10.59; 95% CI: 9.54 to 11.65) **(**Table 2; Fig. 7, Supplementary Table 6).

### State-Specific trends

**Fig. 8:**
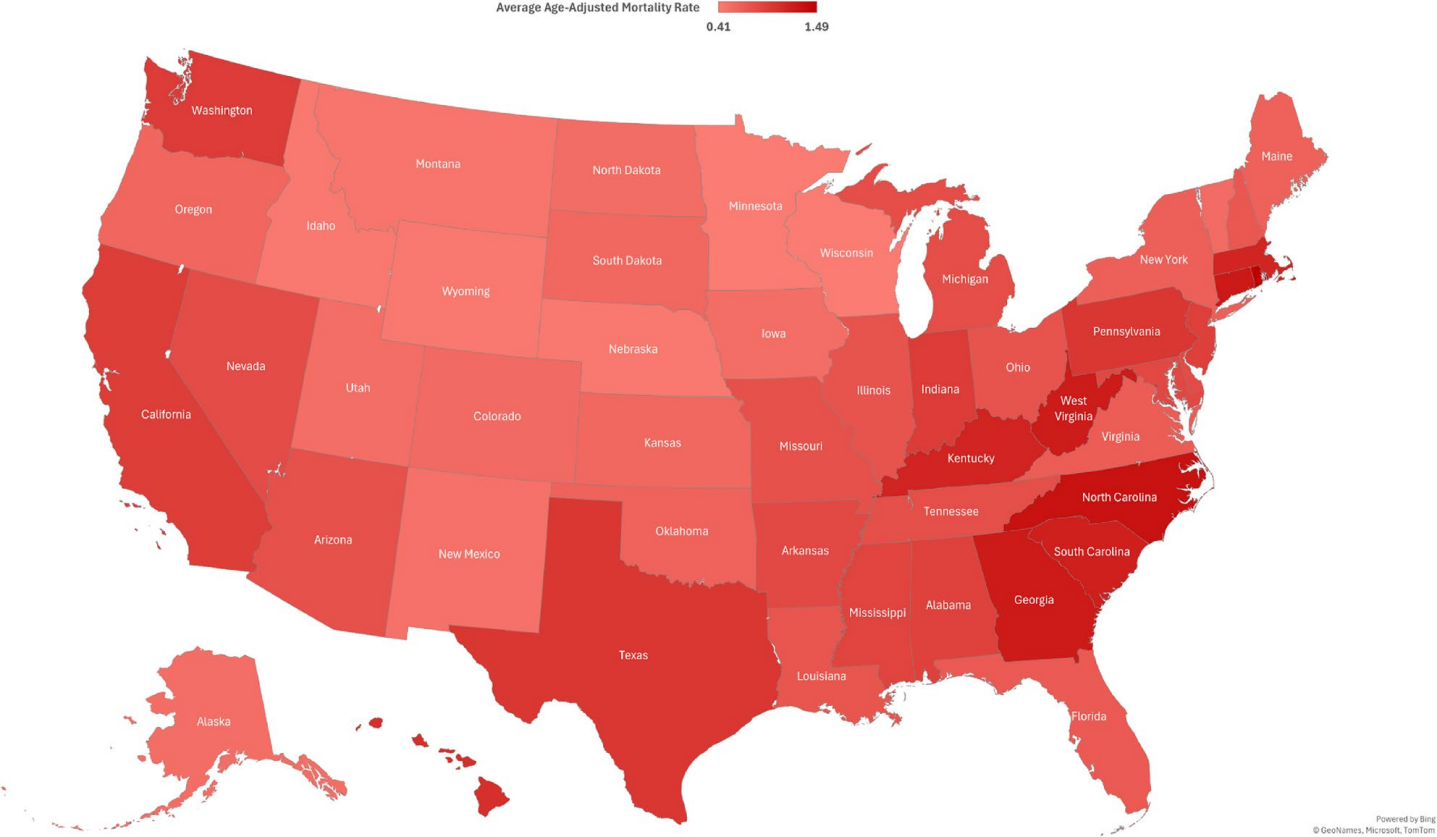
Cardiogenic Shock and Sepsis-Related Average Age-Adjusted Mortality Rates by State, 1999–2023

The state with the highest AAMR is Rhode Island, with a value of 1.68, while the state with the lowest AAMR is Wisconsin at 0.46. States in the 90th percentile for AAMR include Rhode Island, North Carolina, West Virginia, Connecticut, the District of Columbia, and Georgia, all with values at or above 1.31. On the other hand, states in the 10th percentile include Wisconsin, Minnesota, Nebraska, Idaho, Montana, and Wyoming, with values at or below 0.58 **(**Fig. 8, Supplementary Table 7).

## Discussion

CS and sepsis are known to interrelate through shared mechanisms. While both conditions individually cause high mortality rates, coexistence as mixed septic–CS significantly amplifies the risk, with contemporary reports documenting a 63% mortality burden [16]. Moreover, sepsis-associated cardiovascular disease mortality has observed a significant rise in the past two decades, reinforcing the effect of systemic inflammation on cardiovascular compromise [17]. Sepsis is known to precipitate sepsis-induced cardiomyopathy and shock, while patients with pre-existing cardiac dysfunction are specifically more prone to septic infections, creating a vicious cycle [16, 18–20].

When analysed separately, CS mortality rose consistently throughout the study period while overall sepsis-associated mortality remained relatively stable. These findings are further validated by previous studies identifying a similar mortality trend [21, 22]. When considered together, these findings of rising mortality lend support to the hypothesis that interacting pathophysiology of both conditions contributes to excess mortality [17, 23]. 

Our study reports a significant and sustained rise in CS and sepsis-related mortality in the United States from 1999 to 2023. The AAMR surged more than three-fold during the study period, representing a major health crisis. Prior reports also document rising sepsis-related mortality over recent decades, especially in older adults and critically ill populations [24, 25]. When found in conjunction with CS, the death toll continued to rise, despite improvements in cardiovascular critical care such as revascularization, hemodynamic support, and mechanical circulatory devices [26–28]. While advances in critical care, such as sepsis bundles, initially improved survival rates in sepsis patients, recent literature suggests that this progress has plateaued as well [29]. This synergistic interaction highlights the importance of early identification, risk-stratification and tailored management to optimize patient survival.

From 1999 to 2023, our analysis reported AAMRs for CS and sepsis-related deaths to rise sharply from 0.65 to 2.00 per 100,000 population. Overall, mortality showed a stable uptrend, with an AAPC of 5.39%, rising modestly from 1999 to 2009 but surging significantly through 2021. This pattern aligned closely with sepsis-associated cardiovascular disease mortality previously reported [17].

The marked rise observed in sepsis and CS-related deaths during 2020–2021, likely reflects the impact of the COVID-19 pandemic on healthcare dynamics in the U.S. The pandemic had a profound impact on healthcare systems globally, potentially delaying access to timely critical care for CS and sepsis patients, worsening mortality outcomes [30–32]. Additionally, SARS-CoV-2 infection was well-recognized to have secondary bacterial infections and sepsis-like presentations, which may have contributed to higher coding of sepsis and septic shock as an underlying or contributing cause of death [33].

Our study found consistently higher AAMRs among men compared to women throughout the study period. This is consistent with the greater sepsis-associated cardiovascular disease mortality burden identified in men [17]. Prognosis in men is likely influenced by a higher prevalence of comorbid illnesses, smoking and alcohol use, and inherent predisposition for visceral obesity [34]. However, women with CS (whether from acute myocardial infarction or advanced heart failure) were consistently less likely to receive invasive monitoring, early revascularization, or mechanical circulatory support (MCS) [35]. Even after adjusting for confounding factors, women have approximately 10% higher in-hospital mortality compared to men and are significantly less likely to receive MCS [35–37]. These disparities are partly attributable to the greater age and comorbidity burden among women and highlight a pressing need for equitable life-saving care [36].

When analysed separately, men experienced higher mortality than women from both sepsis and CS individually. These findings mirror prior evidence showing greater physiologic instability, immune dysregulation, and subsequent organ failure in critical illness observed among men [38–40]. A prior meta-analysis reported a higher 28-day mortality in women and lower 1-year mortality in women, however overall certainty of evidence was low due to variability in results [35]. On the contrary, our population-level analysis consistently displays a higher mortality among men reflecting an extension of underlying susceptibility rather than a reversal of sex disparities. Overall, sex differences in critical illness mortality outcomes are likely to be context-dependent and influenced by biological factors, co-morbid conditions, treatment response and lifestyle factors.

Among racial groups, Black populations persistently exhibited higher mortality compared to White populations, with the gap widening during certain time intervals. These disparities reinforce past reports of a disproportionately high sepsis-related mortality among Blacks, where structural inequities, differential access to critical care, and a higher baseline burden of comorbidities were major contributors [10, 17, 41–45]. Black and Hispanic patients are consistently reported to have a higher background prevalence of comorbidities like diabetes and chronic kidney disease, but conversely, lower access to invasive monitoring, timely coronary angiography, and advanced mechanical circulatory support (MCS) for CS. Provider-level factors such as implicit bias and patients’ insurance-related barriers contribute to these gaps, making life-saving interventions unfortunately less likely for the minorities [45, 46]. Even when MCS is used, this subgroup is less likely to be offered more advanced therapies such as heart transplantation, largely due to socioeconomic barriers, multiplying mortality risks [42]. For sepsis, socioeconomically disadvantaged patients often present later in the disease course, with more advanced infection and multi-organ dysfunction, due to barriers in accessing timely primary and emergency care [47–49]. Hospital-level factors play a key role: safety-net and rural hospitals, which disproportionately serve Black and Hispanic patients, often have fewer ICU resources, lower nurse-to-patient ratios, and delayed adoption of sepsis bundles [49, 50] These systemic issues collectively explain why Black patients, particularly in low-resource areas, experience higher sepsis mortality, even when adjustments are made for illness severity.

Older adults (≥ 65 years) were reported to experience the highest mortality burden from sepsis and CS, as well as the highest APCs among all age-groups. The rapid rise observed in mortality aligns with the rising rates of hospitalisations and increased susceptibility of healthcare-acquired infections reported in this age group [51, 52]. Moreover, age-related decline in immune and cardiac function is made worse by increasing rates of polypharmacy and sexually transmitted infections (STIs) in the older adult population [53, 54]. This reflects factors like a decreased physiological reserve, frailty and multi-morbidity in this at-risk population [24, 55]. Older individuals are known to have a diminished immune and cardiovascular reserve, leading to poorer pathogenic clearance, exaggerated inflammation, and impaired hemodynamic compensation [56]. In addition, comorbidities such as diabetes, chronic kidney disease and heart failure, further exacerbate vulnerability to severe infection, organ decompensation and shock [57, 58]. Together, these factors contribute to poorer tolerance to aggressive interventions, amplifying the risk of complications and in-hospital death.

Our analysis also reveals a concerning uptrend in young adults (24–44 years). Individuals aged 25–44 years are reported to display sharply rising mortality, aligning with contemporary reports [59–61]. A combination of worsening baseline cardiovascular health and infectious complications leads to the observed trend. The opioid epidemic, in particular, has remarkably increased intravenous drug abuse, raising the incidence of infections such as infective endocarditis, which that precipitate severe sepsis [62]. Similarly, the rising prevalence of cardiovascular risk factors like obesity, diabetes, and hypertension has led to higher rates of heart failure and CS in young and middle-aged adults (45–64 years), suggesting that prevention and early detection strategies should not be confined to the older populations solely [63].

Our analysis of geographical disparities in mortality revealed a higher mortality in rural areas compared to urban counterparts across the two decades, reinforcing the well-documented “rural mortality penalty” [64, 65]. Limited access to healthcare, delays in timely diagnosis and management, physician shortages and higher poverty rates are underlying factors that lead to distinct disparities across geographical margins. Regionally, the Midwest and West demonstrated the highest mortality upsurge, followed by the Southern Census Region, suggesting that both systemic health infrastructure and local practice patterns influence outcomes [66, 67]. Rural hospitals often lack access to PCI-capable (PCI: percutaneous coronary intervention) centers, advanced circulatory support for CS, and standardized sepsis protocols, which results in preventable delays in care. Similarly, the pronounced up-trend in mortality observed in the Southern census region warrants particular attention. This region historically exhibits higher burdens of cardiovascular and metabolic diseases, greater poverty burden and limited access to tertiary healthcare services. Along with this, potential differences in health literacy and preventive care may also contribute to the disproportionate increase in mortality from both cardiogenic shock and sepsis in the Southern region [67, 68].

Interestingly, our analysis demonstrated a higher mortality in the Midwest compared with the Southern census region, however mortality rose in all regions. This divergence from previous AMI-CS (acute myocardial infection - cardiogenic shock) literature likely reflects differences in population structure, with the Midwest exhibiting an older age profile and a greater burden of chronic heart failure and ischemic cardiomyopathy as compared to the Southern Census region. Demographic constraints, such as a lower density of high-volume PCI and advanced cardiac-care centers in rural Midwestern states, may contribute to poorer outcomes. Regional variations in ICD-based cause-of-death documentation may also compound the observed mortality, with under-recognition of cardiac causes documented more frequently in certain Southern states. The persistent rise in mortality observed in Northeastern regions of the country contrasts with prior evidence of declining sepsis-associated cardiovascular disease mortality identified in this region [17]. Higher rates may reflect increased detection and reporting in hospital settings, regional disparities in care, and temporal increases in critical illness severity, requiring further insight.

Closing this gap will require strengthening rural healthcare infrastructure through better referral networks, expanded tele-ICU and transfer services, equitable distribution of MCS services, and consistent adoption of sepsis bundles [69, 70]. Over time, such improvements can help reduce the rural-urban disparity to bring outcomes closer to those achieved in well-resourced urban centers. Moreover, the concentration of deaths in medical facilities underscores the role of hospital-level interventions in improving patient outcomes.

State-level variations in mortality were also notable, with some states like Rhode Island, demonstrating persistently higher AAMRs (1.68) compared to states like Wisconsin (0.46). This likely overlaps regional differences in healthcare access and infrastructure, availability of advanced cardiac-care units and population-based differences in health-seeking behavior across different regions [71, 72].

States with lower reported mortality may also be influenced by underreporting or incomplete death certification, which is unfortunately common in smaller or geographically marginalized healthcare facilities. Standardizing CS and sepsis management, through public health policy reformation and quality improvement initiatives, can help bridge these gaps in future.

### Limitations

Strengths of this study include its large, nationally representative dataset spanning 25 years, enabling reliable temporal and subgroup analyses. However, certain limitations warrant consideration. This study does not take into account changes in coding practices over time, nor does it consider granular clinical variables such as pre-existing patient health profiles, treatment strategies opted or severity of illness. Personal records of patients’ socioeconomic characteristics such as income, insurance status or access to healthcare were lacking, which potentially confound both the incidence and outcomes of all diseases, limiting our ability to fully characterize mortality disparities. Lower mortality rates observed in certain states may partly reflect data limitations rather than true epidemiologic differences. Variability in reporting practices, delays in death certificate updates, or incomplete data submission from smaller healthcare providers could contribute to this pattern. Lastly, race/ethnicity analyses were limited to non-Hispanic White, non-Hispanic Black, and Hispanic populations because reliable age-adjusted mortality rates were unavailable for other racial/ethnic groups in the CDC WONDER database.

Future research incorporating socioeconomic and healthcare access data could better explain the documented disparities. Similarly, prospective studies aimed at evaluating targeted interventions such as regionalized critical care networks, tele-ICU programs and equity-focused sepsis bundles, may improve overall mortality outcomes.

## Conclusion

Despite advances in critical care, high mortality rates from CS and sepsis persist, highlighting ongoing challenges in optimizing management. Our reports reveal CS and sepsis-related mortality in the United States increased substantially from 1999 to 2023, with a disproportionately higher burden among men, Black individuals, older adults, rural populations, Midwest and Southern Census regions.

Equitable access to care, tailored prevention, early identification and effective public health strategies are the need of the hour. Future research is also required to further explore factors underlying the documented disparities, improve risk-stratification and curate integrative therapies to curb rising trends from both the diseases.

Supplementary Materials.

Contains supplemental tables and supplemental figures referenced in the manuscript as an additional file.

## Supplementary Information

Supplementary Material 1.

## Data Availability

The datasets generated and/or analysed during the current study are available on the Centers for Disease Control and Prevention Public Database, [[https://wonder.cdc.gov/mcd-icd10.html](https:/wonder.cdc.gov/mcd.html)].

## Abbreviations

CS: Cardiogenic shock
AAMR: Age-adjusted mortality rate
CMR: Crude Mortality Rate
AAPC: Average annual percentage change
APC: Annual percentage change
