# Supplementary material for: Nationwide trends and disparities in deaths following cardiogenic shock and sepsis in the United States (1999–2023): insights from the CDC WONDER database
Source: BMC Cardiovasc Disord. 2025 Dec 6;26:24. doi: 10.1186/s12872-025-05402-3 (PMC12797607; doi:10.1186/s12872-025-05402-3)
Supplement: Supplementary file 1 — Supplementary Material 1. [file 12872_2025_5402_MOESM1_ESM.docx]

**SUPPLEMENTARY TABLES**

**Supplemental Table 1:** Age-adjusted mortality rates per 100,000 for Cardiogenic Shock alone and Sepsis alone among adults aged ≥25 in the United States, 1999 to 2023.

**Supplemental Table 2:** Overall and sex‐stratified Cardiogenic Shock and Sepsis–related age-adjusted mortality rates per 100,000 among adults aged ≥25 in the United States, 1999 to 2023

**Supplemental Table 3:** Cardiogenic Shock and Sepsis-related age-adjusted mortality rates per 100,000 stratified by race/ethnicity among adults aged ≥25 in the United States, 1999 to 2023.

**Supplemental Table 4:** Cardiogenic Shock and Sepsis–related crude mortality rates per 100,000 stratified by age group among adults aged ≥25 in the United States, 1999 to 2023.

**Supplemental Table 5:** Cardiogenic Shock and Sepsis-related age-adjusted mortality rates per 100,000 stratified by urban-rural classification among adults aged ≥25 in the United States, 1999 to 2023

**Supplemental Table 6:** Cardiogenic Shock and Sepsis-related age-adjusted mortality rates per 100,000 stratified by U.S. census region among adults aged ≥25 in the United States, 1999 to 2023

**Supplemental Table 7:** Cardiogenic Shock and Sepsis-related age-adjusted mortality rates per 100,000 stratified by state among adults aged ≥25 in the United States, 1999 to 2023.

**Supplemental Table 8:** Cardiogenic Shock and Sepsis-related deaths stratified by place of death among adults aged ≥25 in the United States, 1999 to 2023

**Supplemental Table 1:** Age-adjusted mortality rates per 100,000 for Cardiogenic Shock alone and Sepsis alone among adults aged ≥25 in the United States, 1999–2023.

| **Year** | **Age-Adjusted Mortality Rate (95% CI)** | | |
| --- | --- | --- | --- |
|  | **Overall (Cardiogenic Shock and Sepsis Combined)** | **Cardiogenic Shock (Alone)** | **Sepsis (Alone)** |
| **1999** | 0.65 (0.61 – 0.69) | 10.72 (10.57-10.87) | 77.83 (77.42-78.24) |
| **2000** | 0.58 (0.54 – 0.61) | 9.82 (9.68-9.97) | 75.99 (75.58-76.39) |
| **2001** | 0.54 (0.51 – 0.57) | 8.71 (8.57-8.85) | 76.12 (75.72-76.52) |
| **2002** | 0.59 (0.55 – 0.62) | 8.34 (8.20-8.47) | 76.69 (76.29-77.09) |
| **2003** | 0.52 (0.49 – 0.55) | 7.70 (7.57-7.82) | 76.52 (76.12-76.91) |
| **2004** | 0.56 (0.53 – 0.60) | 7.40 (7.28-7.52) | 75.53 (75.14-75.92) |
| **2005** | 0.57 (0.54 – 0.61) | 7.08 (6.96-7.20) | 77.60 (77.21-77.99) |
| **2006** | 0.55 (0.52 – 0.58) | 6.94 (6.83-7.06) | 76.01 (75.62-76.39) |
| **2007** | 0.57 (0.54 – 0.60) | 6.79 (6.67-6.90) | 74.56 (74.19-74.94) |
| **2008** | 0.63 (0.60 – 0.67) | 7.00 (6.88-7.11) | 76.32 (75.94-76.70) |
| **2009** | 0.62 (0.58 – 0.65) | 6.80 (6.69-6.92) | 73.98 (73.61-74.35) |
| **2010** | 0.66 (0.63 – 0.70) | 7.00 (6.88-7.11) | 73.80 (73.43-74.16) |
| **2011** | 0.72 (0.68 – 0.75) | 7.25 (7.14-7.36) | 74.30 (73.94-74.67) |
| **2012** | 0.75 (0.71 – 0.78) | 7.50 (7.38-7.61) | 72.66 (72.30-73.01) |
| **2013** | 0.89 (0.85 – 0.93) | 7.76 (7.65-7.88) | 74.76 (74.40-75.11) |
| **2014** | 1.02 (0.98 – 1.06) | 8.45 (8.33-8.57) | 76.63 (76.27-76.98) |
| **2015** | 1.15 (1.11 – 1.20) | 9.34 (9.21-9.46) | 80.31 (79.94-80.67) |
| **2016** | 1.30 (1.26 – 1.35) | 10.00 (9.87-10.13) | 79.86 (79.50-80.22) |
| **2017** | 1.36 (1.32 – 1.41) | 10.50 (10.38-10.63) | 80.37 (80.01-80.73) |
| **2018** | 1.50 (1.45 – 1.55) | 11.29 (11.16-11.42) | 79.55 (79.20-79.90) |
| **2019** | 1.56 (1.51 – 1.61) | 11.98 (11.85-12.12) | 76.24 (75.90-76.58) |
| **2020** | 1.74 (1.69 – 1.79) | 12.71 (12.58-12.85) | 90.47 (90.10-90.84) |
| **2021** | 2.04 (1.99 – 2.10) | 14.56 (14.41-14.70) | 99.38 (98.99-99.77) |
| **2022** | 2.03 (1.98 – 2.09) | 14.22 (14.08-14.37) | 89.15 (88.79-89.51) |
| **2023** | 2.01 (1.96 – 2.06) | 14.23 (14.08-14.37) | 81.79 (81.44-82.13) |

**Supplemental Table 2:** Overall and sex‐stratified Cardiogenic Shock and Sepsis–related age-adjusted mortality rates per 100,000 among adults aged ≥25 in the United States, 1999 to 2023

| **Year** | **Age-Adjusted Mortality Rate (95% CI)** | | |
| --- | --- | --- | --- |
|  | **Overall (Cardiogenic Shock and Sepsis Combined)** | **Female** | **Male** |
| **1999** | 0.65 (0.61 – 0.69) | 0.52 (0.47–0.56) | 0.81 (0.74–0.87) |
| **2000** | 0.58 (0.54 – 0.61) | 0.49 (0.44–0.53) | 0.73 (0.67–0.79) |
| **2001** | 0.54 (0.51 – 0.57) | 0.41 (0.37–0.45) | 0.67 (0.61–0.72) |
| **2002** | 0.59 (0.55 – 0.62) | 0.47 (0.43–0.51) | 0.71 (0.65–0.77) |
| **2003** | 0.52 (0.49 – 0.55) | 0.41 (0.37–0.45) | 0.69 (0.63–0.75) |
| **2004** | 0.56 (0.53 – 0.60) | 0.49 (0.44–0.53) | 0.66 (0.60–0.72) |
| **2005** | 0.57 (0.54 – 0.61) | 0.50 (0.46–0.54) | 0.72 (0.66–0.78) |
| **2006** | 0.55 (0.52 – 0.58) | 0.50 (0.45–0.54) | 0.69 (0.63–0.74) |
| **2007** | 0.57 (0.54 – 0.60) | 0.45 (0.41–0.49) | 0.69 (0.63–0.74) |
| **2008** | 0.63 (0.60 – 0.67) | 0.51 (0.47–0.55) | 0.78 (0.73–0.84) |
| **2009** | 0.62 (0.58 – 0.65) | 0.50 (0.46–0.54) | 0.77 (0.71–0.83) |
| **2010** | 0.66 (0.63 – 0.70) | 0.50 (0.46–0.54) | 0.87 (0.81–0.93) |
| **2011** | 0.72 (0.68 – 0.75) | 0.57 (0.53–0.62) | 0.88 (0.82–0.94) |
| **2012** | 0.75 (0.71 – 0.78) | 0.61 (0.56–0.65) | 0.94 (0.87–1.00) |
| **2013** | 0.89 (0.85 – 0.93) | 0.67 (0.62–0.72) | 1.14 (1.07–1.21) |
| **2014** | 1.02 (0.98 – 1.06) | 0.81 (0.76–0.86) | 1.26 (1.19–1.33) |
| **2015** | 1.15 (1.11 – 1.20) | 0.92 (0.86–0.97) | 1.46 (1.38–1.53) |
| **2016** | 1.30 (1.26 – 1.35) | 0.99 (0.93–1.04) | 1.71 (1.63–1.79) |
| **2017** | 1.36 (1.32 – 1.41) | 1.05 (0.99–1.10) | 1.80 (1.72–1.88) |
| **2018** | 1.50 (1.45 – 1.55) | 1.12 (1.06–1.17) | 1.98 (1.89–2.06) |
| **2019** | 1.56 (1.51 – 1.61) | 1.15 (1.09–1.20) | 2.07 (1.98–2.15) |
| **2020** | 1.74 (1.69 – 1.79) | 1.31 (1.25–1.37) | 2.29 (2.20–2.37) |
| **2021** | 2.04 (1.99 – 2.10) | 1.53 (1.46–1.59) | 2.64 (2.55–2.73) |
| **2022** | 2.03 (1.98 – 2.09) | 1.47 (1.54 - 1.60) | 2.58 (2.49–2.68) |
| **2023** | 2.01 (1.96 – 2.06) | 1.47 (1.53 - 1.60) | 2.60 (2.51–2.69) |

**Supplemental Table 3:** Cardiogenic Shock and Sepsis-related age-adjusted mortality rates per 100,000 stratified by race/ethnicity among adults aged ≥25 in the United States, 1999 to 2023.

| **Year** | **Age-Adjusted Mortality Rate (95% CI)** | | |
| --- | --- | --- | --- |
|  | **NH Black or African American** | **NH White** | **Hispanic or Latino** |
| **1999** | 1.24 (1.06–1.41) | 0.58 (0.54–0.62) | 0.84 (0.65–1.06) |
| **2000** | 1.02 (0.86–1.18) | 0.55 (0.51–0.58) | 0.73 (0.56–0.93) |
| **2001** | 1.07 (0.91–1.24) | 0.49 (0.45–0.52) | 0.63 (0.48–0.81) |
| **2002** | 0.94 (0.79–1.09) | 0.56 (0.52–0.60) | 0.70 (0.54–0.88) |
| **2003** | 0.84 (0.70–0.98) | 0.50 (0.46–0.53) | 0.55 (0.41–0.72) |
| **2004** | 0.89 (0.74–1.03) | 0.53 (0.49–0.56) | 0.56 (0.43–0.72) |
| **2005** | 0.93 (0.78–1.07) | 0.52 (0.49–0.56) | 0.59 (0.46–0.75) |
| **2006** | 0.92 (0.78–1.06) | 0.52 (0.49–0.56) | 0.61 (0.48–0.77) |
| **2007** | 0.97 (0.82–1.11) | 0.52 (0.48–0.55) | 0.65 (0.51–0.80) |
| **2008** | 0.96 (0.82–1.10) | 0.58 (0.54–0.61) | 0.68 (0.54–0.82) |
| **2009** | 1.08 (0.93–1.22) | 0.56 (0.53–0.59) | 0.55 (0.43–0.68) |
| **2010** | 1.00 (0.86–1.14) | 0.63 (0.59–0.67) | 0.60 (0.48–0.73) |
| **2011** | 1.22 (1.07–1.38) | 0.66 (0.63–0.70) | 0.61 (0.49–0.73) |
| **2012** | 1.33 (1.17–1.49) | 0.69 (0.65–0.73) | 0.76 (0.63–0.89) |
| **2013** | 1.41 (1.25–1.57) | 0.82 (0.78–0.86) | 0.65 (0.53–0.77) |
| **2014** | 1.78 (1.60–1.95) | 0.92 (0.88–0.97) | 0.78 (0.65–0.90) |
| **2015** | 1.93 (1.75–2.11) | 1.07 (1.02–1.12) | 1.01 (0.88–1.15) |
| **2016** | 2.04 (1.86–2.22) | 1.22 (1.17–1.27) | 1.14 (1.00–1.28) |
| **2017** | 2.24 (2.05–2.42) | 1.28 (1.23–1.33) | 1.42 (1.26–1.57) |
| **2018** | 2.46 (2.27–2.65) | 1.36 (1.31–1.41) | 1.44 (1.28–1.59) |
| **2019** | 2.64 (2.44–2.83) | 1.44 (1.38–1.49) | 1.45 (1.30–1.60) |
| **2020** | 2.91 (2.71–3.11) | 1.60 (1.54–1.65) | 1.78 (1.62–1.94) |
| **2021** | 3.39 (3.17–3.61) | 1.90 (1.84–1.96) | 2.00 (1.83–2.17) |
| **2022** | 3.49 (3.27–3.71) | 1.85 (1.79–1.91) | 2.04 (1.87–2.21) |
| **2023** | 3.58 (3.36–3.80) | 1.82 (1.77–1.88) | 1.90 (1.74–2.06) |

NH = non-Hispanic

**Supplemental Table 4:** Cardiogenic Shock and Sepsis–related crude mortality rates per 100,000 stratified by age group among adults aged ≥25 in the United States, 1999 to 2023.

| **Year** | **Crude Mortality Rate (95% CI)** | | |
| --- | --- | --- | --- |
|  | **Young adults** | **Middle-aged adults** | **Older adults** |
| **1999** | 0.04 (0.03–0.05) | 0.37 (0.32–0.42) | 2.52 (2.35–2.69) |
| **2000** | 0.04 (0.03–0.06) | 0.37 (0.32–0.41) | 2.20 (2.05–2.36) |
| **2001** | 0.04 (0.03–0.06) | 0.32 (0.28–0.37) | 2.10 (1.95–2.25) |
| **2002** | 0.05 (0.03–0.06) | 0.38 (0.33–0.43) | 2.22 (2.06–2.37) |
| **2003** | 0.03 (0.02–0.05) | 0.33 (0.29–0.38) | 2.12 (1.97–2.27) |
| **2004** | 0.06 (0.04–0.07) | 0.35 (0.31–0.40) | 2.14 (1.99–2.29) |
| **2005** | 0.05 (0.04–0.07) | 0.35 (0.31–0.39) | 2.22 (2.06–2.37) |
| **2006** | 0.05 (0.04–0.07) | 0.39 (0.34–0.43) | 2.08 (1.93–2.23) |
| **2007** | 0.05 (0.04–0.07) | 0.32 (0.28–0.36) | 2.25 (2.09–2.40) |
| **2008** | 0.06 (0.05–0.08) | 0.44 (0.40–0.49) | 2.31 (2.16–2.46) |
| **2009** | 0.07 (0.06–0.09) | 0.46 (0.42–0.51) | 2.22 (2.07–2.37) |
| **2010** | 0.07 (0.05–0.09) | 0.46 (0.42–0.51) | 2.45 (2.30–2.60) |
| **2011** | 0.09 (0.07–0.11) | 0.49 (0.44–0.54) | 2.60 (2.44–2.75) |
| **2012** | 0.07 (0.05–0.09) | 0.58 (0.53–0.64) | 2.71 (2.55–2.86) |
| **2013** | 0.12 (0.09–0.14) | 0.67 (0.62–0.73) | 3.04 (2.88–3.20) |
| **2014** | 0.12 (0.10–0.15) | 0.75 (0.69–0.81) | 3.53 (3.36–3.70) |
| **2015** | 0.13 (0.11–0.16) | 0.94 (0.87–1.00) | 3.93 (3.76–4.11) |
| **2016** | 0.17 (0.14–0.20) | 1.01 (0.95–1.08) | 4.49 (4.30–4.68) |
| **2017** | 0.17 (0.15–0.20) | 1.08 (1.01–1.15) | 4.72 (4.53–4.91) |
| **2018** | 0.20 (0.17–0.23) | 1.27 (1.19–1.34) | 5.02 (4.83–5.21) |
| **2019** | 0.20 (0.17–0.23) | 1.31 (1.23–1.39) | 5.25 (5.05–5.44) |
| **2020** | 0.24 (0.21–0.27) | 1.54 (1.46–1.63) | 5.70 (5.50–5.90) |
| **2021** | 0.30 (0.26–0.33) | 1.76 (1.67–1.85) | 6.61 (6.40–6.83) |
| **2022** | 0.27 (0.24–0.30) | 1.69 (1.60–1.78) | 6.69 (6.48–6.90) |
| **2023** | 0.26 (0.23–0.29) | 1.64 (1.55–1.73) | 6.70 (6.49–6.91) |

**Supplemental Table 5:** Cardiogenic Shock and Sepsis-related age-adjusted mortality rates per 100,000 stratified by urban-rural classification among adults aged ≥25 in the United States, 1999 to 2023

| **Year** | **Age-Adjusted Mortality Rate (95% CI)** | |
| --- | --- | --- |
|  | **Urban Areas (Metropolitan)** | **Rural Areas (Non-metropolitan)** |
| **1999** | 0.65 (0.61–0.69) | 0.60 (0.52–0.69) |
| **2000** | 0.60 (0.56–0.64) | 0.52 (0.44–0.60) |
| **2001** | 0.57 (0.53–0.61) | 0.46 (0.38–0.53) |
| **2002** | 0.60 (0.56–0.64) | 0.56 (0.48–0.64) |
| **2003** | 0.54 (0.50–0.57) | 0.47 (0.39–0.54) |
| **2004** | 0.57 (0.53–0.60) | 0.54 (0.46–0.62) |
| **2005** | 0.58 (0.54–0.62) | 0.52 (0.44–0.60) |
| **2006** | 0.55 (0.52–0.59) | 0.55 (0.48–0.63) |
| **2007** | 0.60 (0.56–0.64) | 0.47 (0.39–0.54) |
| **2008** | 0.63 (0.59–0.66) | 0.57 (0.49–0.65) |
| **2009** | 0.62 (0.59–0.66) | 0.59 (0.51–0.66) |
| **2010** | 0.66 (0.62–0.70) | 0.65 (0.57–0.74) |
| **2011** | 0.71 (0.67–0.75) | 0.75 (0.66–0.84) |
| **2012** | 0.75 (0.71–0.79) | 0.72 (0.64–0.80) |
| **2013** | 0.87 (0.83–0.91) | 1.00 (0.89–1.10) |
| **2014** | 1.01 (0.96–1.06) | 1.10 (0.99–1.21) |
| **2015** | 1.14 (1.09–1.19) | 1.27 (1.16–1.39) |
| **2016** | 1.26 (1.21–1.30) | 1.50 (1.37–1.62) |
| **2017** | 1.34 (1.29–1.39) | 1.47 (1.36–1.59) |
| **2018** | 1.47 (1.42–1.53) | 1.71 (1.58–1.84) |
| **2019** | 1.54 (1.49–1.60) | 1.71 (1.58–1.83) |
| **2020** | 1.70 (1.65–1.76) | 2.03 (1.88–2.17) |

**Supplemental Table 6:** Cardiogenic Shock and Sepsis-related age-adjusted mortality rates per 100,000 stratified by U.S. census region among adults aged ≥25 in the United States, 1999 to 2023

| **Year** | **Age-Adjusted Mortality Rate (95% CI)** | | | |
| --- | --- | --- | --- | --- |
|  | **Northeast** | **Midwest** | **South** | **West** |
| **1999** | 0.80 (0.71–0.89) | 0.54 (0.47–0.61) | 0.67 (0.60–0.74) | 0.59 (0.51–0.67) |
| **2000** | 0.69 (0.60–0.77) | 0.49 (0.42–0.56) | 0.61 (0.55–0.67) | 0.48 (0.41–0.55) |
| **2001** | 0.62 (0.54–0.70) | 0.44 (0.38–0.51) | 0.55 (0.49–0.61) | 0.53 (0.45–0.60) |
| **2002** | 0.66 (0.58–0.74) | 0.44 (0.37–0.50) | 0.64 (0.58–0.71) | 0.56 (0.48–0.63) |
| **2003** | 0.61 (0.53–0.69) | 0.47 (0.40–0.53) | 0.53 (0.48–0.59) | 0.52 (0.45–0.60) |
| **2004** | 0.66 (0.58–0.75) | 0.44 (0.38–0.51) | 0.55 (0.49–0.60) | 0.57 (0.49–0.64) |
| **2005** | 0.66 (0.58–0.74) | 0.48 (0.41–0.54) | 0.56 (0.51–0.62) | 0.58 (0.50–0.65) |
| **2006** | 0.72 (0.63–0.80) | 0.41 (0.35–0.47) | 0.60 (0.54–0.65) | 0.48 (0.42–0.55) |
| **2007** | 0.74 (0.66–0.83) | 0.42 (0.36–0.48) | 0.55 (0.50–0.61) | 0.58 (0.50–0.65) |
| **2008** | 0.76 (0.68–0.85) | 0.54 (0.47–0.61) | 0.60 (0.54–0.65) | 0.62 (0.54–0.69) |
| **2009** | 0.67 (0.59–0.75) | 0.54 (0.47–0.61) | 0.62 (0.57–0.68) | 0.61 (0.54–0.69) |
| **2010** | 0.79 (0.70–0.87) | 0.58 (0.51–0.65) | 0.63 (0.57–0.69) | 0.66 (0.59–0.74) |
| **2011** | 0.81 (0.72–0.89) | 0.59 (0.52–0.66) | 0.72 (0.66–0.78) | 0.72 (0.65–0.80) |
| **2012** | 0.74 (0.66–0.82) | 0.62 (0.55–0.69) | 0.86 (0.79–0.92) | 0.75 (0.67–0.83) |
| **2013** | 0.99 (0.89–1.08) | 0.69 (0.61–0.76) | 0.97 (0.90–1.04) | 0.80 (0.73–0.88) |
| **2014** | 1.06 (0.96–1.16) | 0.81 (0.73–0.89) | 1.16 (1.09–1.23) | 0.90 (0.82–0.98) |
| **2015** | 1.13 (1.03–1.23) | 0.95 (0.87–1.04) | 1.33 (1.25–1.40) | 1.08 (0.99–1.17) |
| **2016** | 1.34 (1.23–1.45) | 1.06 (0.97–1.15) | 1.42 (1.35–1.50) | 1.29 (1.19–1.39) |
| **2017** | 1.40 (1.29–1.51) | 1.16 (1.07–1.26) | 1.52 (1.44–1.60) | 1.31 (1.21–1.40) |
| **2018** | 1.48 (1.37–1.60) | 1.29 (1.19–1.39) | 1.70 (1.62–1.78) | 1.37 (1.27–1.46) |
| **2019** | 1.46 (1.35–1.57) | 1.41 (1.31–1.51) | 1.72 (1.63–1.80) | 1.56 (1.46–1.66) |
| **2020** | 1.64 (1.53–1.76) | 1.54 (1.44–1.65) | 1.84 (1.76–1.93) | 1.77 (1.66–1.88) |
| **2021** | 1.94 (1.81–2.06) | 1.68 (1.57–1.79) | 2.18 (2.09–2.28) | 2.24 (2.12–2.36) |
| **2022** | 1.95 (1.83–2.08) | 1.73 (1.62–1.84) | 2.08 (1.99–2.17) | 2.18 (2.06–2.30) |
| **2023** | 2.03 (1.91–2.16) | 1.74 (1.63–1.85) | 2.03 (1.95–2.12) | 2.19 (2.07–2.30) |

**Supplemental Table 7:** Cardiogenic Shock and Sepsis-related age-adjusted mortality rates per 100,000 stratified by state among adults aged ≥25 in the United States, 1999 to 2023.

| **State** | **Average Age-Adjusted Mortality Rate (95% CI)** |
| --- | --- |
| **Alabama** | 0.98 (0.91–1.05) |
| **Alaska** | 0.73 (0.53–0.97) |
| **Arizona** | 0.83 (0.78–0.89) |
| **Arkansas** | 0.94 (0.85–1.03) |
| **California** | 0.98 (0.95–1.00) |
| **Colorado** | 0.63 (0.56–0.69) |
| **Connecticut** | 1.36 (1.27–1.45) |
| **Delaware** | 0.98 (0.82–1.15) |
| **District of Columbia** | 1.32 (1.07–1.56) |
| **Florida** | 0.72 (0.70–0.75) |
| **Georgia** | 1.31 (1.25–1.37) |
| **Hawaii** | 1.22 (1.07–1.37) |
| **Idaho** | 0.53 (0.43–0.62) |
| **Illinois** | 0.79 (0.75–0.83) |
| **Indiana** | 1.03 (0.96–1.09) |
| **Iowa** | 0.59 (0.53–0.66) |
| **Kansas** | 0.66 (0.58–0.73) |
| **Kentucky** | 1.26 (1.17–1.34) |
| **Louisiana** | 0.80 (0.74–0.87) |
| **Maine** | 0.75 (0.63–0.86) |
| **Maryland** | 0.93 (0.86–0.99) |
| **Massachusetts** | 1.23 (1.16–1.30) |
| **Michigan** | 0.86 (0.81–0.90) |
| **Minnesota** | 0.46 (0.41–0.51) |
| **Mississippi** | 0.99 (0.89–1.08) |
| **Missouri** | 0.84 (0.78–0.90) |
| **Montana** | 0.58 (0.47–0.70) |
| **Nebraska** | 0.51 (0.43–0.60) |
| **Nevada** | 0.92 (0.82–1.02) |
| **New Hampshire** | 0.84 (0.72–0.97) |
| **New Jersey** | 0.98 (0.93–1.04) |
| **New Mexico** | 0.59 (0.50–0.68) |
| **New York** | 0.68 (0.65–0.71) |
| **North Carolina** | 1.39 (1.33–1.45) |
| **North Dakota** | 0.69 (0.54–0.86) |
| **Ohio** | 0.79 (0.75–0.83) |
| **Oklahoma** | 0.68 (0.62–0.75) |
| **Oregon** | 0.66 (0.59–0.72) |
| **Pennsylvania** | 1.05 (1.01–1.09) |
| **Rhode Island** | 1.68 (1.49–1.87) |
| **South Carolina** | 1.29 (1.21–1.38) |
| **South Dakota** | 0.72 (0.58–0.88) |
| **Tennessee** | 0.85 (0.80–0.91) |
| **Texas** | 1.05 (1.01–1.08) |
| **Utah** | 0.62 (0.53–0.71) |
| **Vermont** | 0.70 (0.55–0.87) |
| **Virginia** | 0.73 (0.68–0.78) |
| **Washington** | 1.03 (0.96–1.09) |
| **West Virginia** | 1.36 (1.24–1.49) |
| **Wisconsin** | 0.46 (0.41–0.50) |
| **Wyoming** | 0.58 (0.43–0.78) |

**Supplemental Table 8:** Cardiogenic Shock and Sepsis-related deaths stratified by place of death among adults aged ≥25 in the United States, 1999 to 2023

| **Place of Death** | **Deaths** | **% of Total Deaths** |
| --- | --- | --- |
| **Medical Facility - Inpatient** | 56670 | 94.61% |
| **Medical Facility - Outpatient or ER** | 1402 | 2.34% |
| **Medical Facility - Dead on Arrival** | 25 | 0.04% |
| **Medical Facility - Status unknown** | 26 | 0.04% |
| **Decedent's home** | 368 | 0.61% |
| **Hospice facility** | 454 | 0.76% |
| **Nursing home/long term care** | 620 | 1.04% |
| **Other** | 201 | 0.34% |
| **Unknown** | 132 | 0.22% |
| **Total** | **59898** | **100.00%** |
